# What evidence exists on birds and mammals' biodiversity in the Brazilian Atlantic Forest (BAF) agricultural ecosystems? A systematic map protocol

**DOI:** 10.1186/s13750-024-00327-4

**Published:** 2024-03-16

**Authors:** Fábio Casallanovo, Gustavo Souza Santos, Ana Paola Cione

**Affiliations:** Syngenta Proteção de Cultivos Ltda, São Paulo, São Paulo Brazil

**Keywords:** Avifauna, Mastofauna, Brazilian Atlantic Forest, Agricultural ecosystems

## Abstract

**Background:**

Brazil has one of the planet's greatest biodiversity, with over 20% of the world’s total species. The Brazilian Atlantic Forest (BAF) spans 17 Brazilian states, making it the third-largest biome in Brazil. The BAF is composed of a range of ecological formations, with climatic conditions and landscape diversity that directly contribute to the different structures of the forest. The fragmentation of the original habitats, mainly due to anthropogenic activities, is one of the main causes of biodiversity loss, causing the decline and habitat loss for several species, including birds and mammals. While there has been extensive research on species diversity in forest fragments or protected areas, there is a lack of basic research in agricultural ecosystems. Only 1.5% of the studies on bird occurrence data in the BAF were reported from pasture habitats and 1.4% from exotic tree plantations. To address this gap, the present systematic map protocol aims to carry out a bibliographic survey on the presence of birds and mammals in agricultural landscapes and its adjacent areas of natural vegetation to describe the prevalence of species across different (semi-)natural and anthropogenic habitat types. Collecting this data is important to support environmental management policies to preserve biodiversity in these areas.

**Methods:**

We will conduct a systematic literature review on the biodiversity of birds and mammals in agricultural landscapes within the Brazilian Atlantic Forest as well as adjacent areas of natural vegetation. Our search will cover the following databases, without limiting the year of publication: Web of Knowledge, Scopus, and PubMed. We will also include grey literature such as dissertations and theses, performing the search at the “*Biblioteca Digital Brasileira*” database. The results will be screened for relevance based on predefined criteria. The screening process will take place in two stages: firstly, the articles will be screened by title and abstract, and then the eligible articles will be screened in full text. Only articles that meet the eligibility criteria will proceed to data extraction. The extracted data will provide the elements to build a systematic map.

**Supplementary Information:**

The online version contains supplementary material available at 10.1186/s13750-024-00327-4.

## Background

Brazil has one of the planet's greatest biodiversity, with over 20% of the world's species spread in the six existing biomes [1]. The Brazilian Atlantic Forest (BAF) is the third largest biome in Brazil, extending across 17 of the nation's 26 states. This area is recognized as one of Earth’s Biodiversity Hotspots due to its high diversity and endemism [2, 3]. The BAF comprises a wide range of distinct ecological formations, with climatic types and landscape diversity that directly contribute to the different structures of the forest [4]. Nonetheless, this biome is threatened because of its proximity to highly populated areas, with only 12.4% of preserved forest remaining [5].

Habitat fragmentation, caused by urbanization, agricultural expansion, and livestock production, constitutes a primary catalyst for the decline in biodiversity 19 [6]. The resultant formation of forest edges due to such fragmentation promotes changes in the ecosystem's physical, chemical, and biological parameters [7]. Consequently, these fragmented habitats have gained attention in research on species occurrence and diversity [8, 9] [21]. Despite the substantial body of research concerning species diversity within forest fragments and protected areas, there is a lack of basic research in human-modified landscapes [10], which include plantations, agricultural fields, tree crops, and pastures. This is particularly true within regions recognized as biodiversity hotspots, such as the Brazilian Atlantic Forest [11].

Data from Mapbiomas 1 indicate that in the Atlantic Forest Biome, as of 2022, only 27.4% of the land is forested. In contrast, over 65% has been altered by anthropogenic activities: pasture (41%), agriculture (27%), a mosaic of land uses (26%), and forestry (6%). Despite the significance of these human-altered landscapes, only 1.5% of bird occurrence studies in the BAF have been conducted in pasture habitats and only 1.4% in exotic tree plantations [11]. In this context, investigating biodiversity within anthropogenically altered landscapes is crucial for enhancing our comprehension of species' responses to human-induced modifications and informing environmental management policies to conserve biodiversity in these regions [12].

Some species may use crop fields as habitat or occasional visitors through spillover [6, 13, 14]. Therefore, birds and mammals could be models to study the influence of landscape structure and matrix composition on cross-habitat spillover into agricultural matrices [6], offering a comprehensive understanding of species' occurrence and their interaction with the landscape. These data can also be used to build a database to understand the biodiversity shifts in anthropogenic areas and their interactions with agricultural landscapes.

**Fig. 1:**
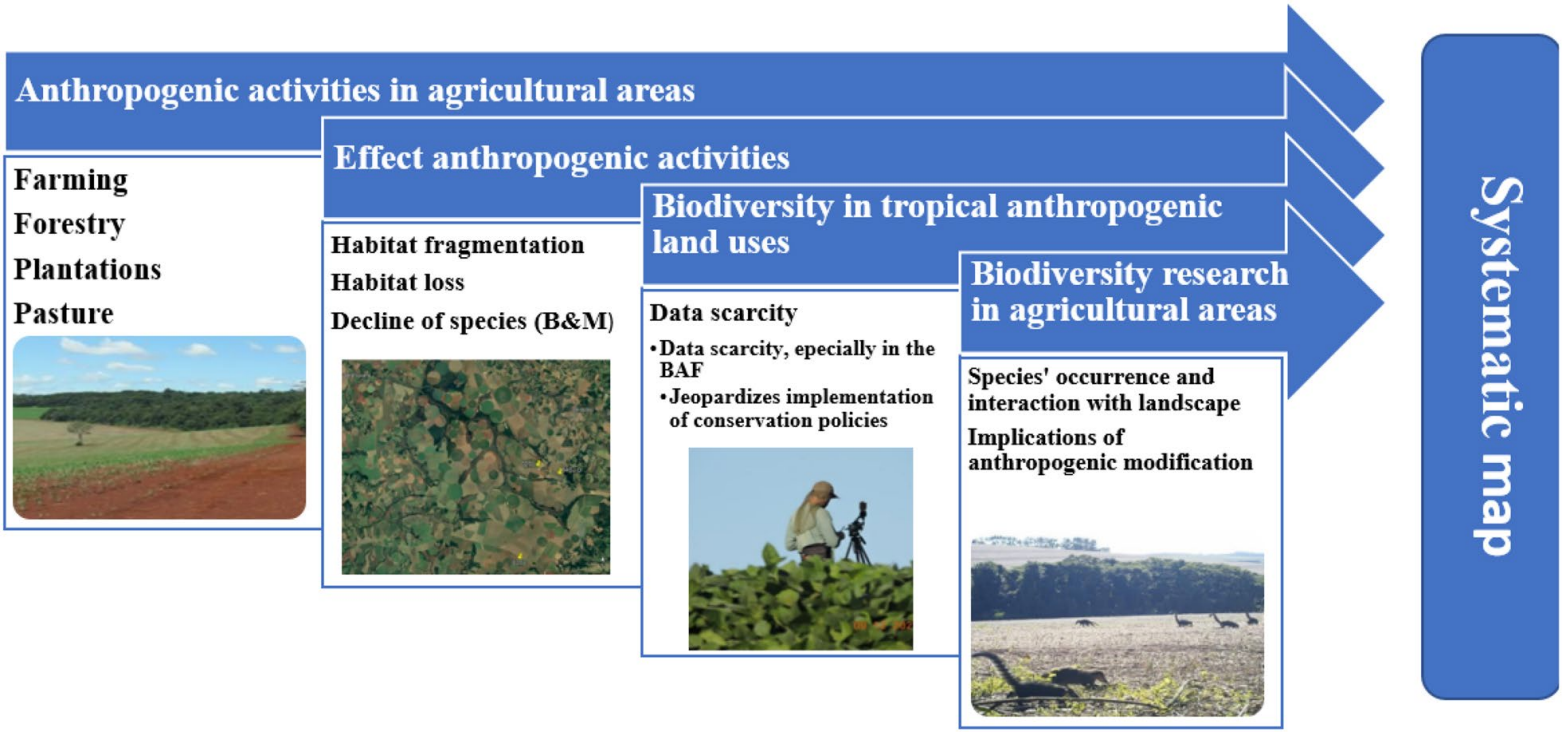
Conceptual model representing the theory of change and the need for a systematic map

On top of that, as summarized in Fig. 1, we intend to build a systematic map that will pinpoint the current knowledge gap about the presence of avian and mammalian species within the agricultural matrix of the Brazilian Atlantic Forest (BAF). The systematic map aims to (a) collate and disseminate information concerning the species of most significant relevance (focus species) within agricultural fields and their adjacent environs of natural vegetation and (b) aid in the selection of representative focal species for environmental risk assessments. Altogether, the assimilation of these data will contribute to construct a comprehensive systematic map, which may help regulators formulate mitigation strategies and conservation policies.

### Stakeholder engagement

Not applicable (N/A).

Only the authors were engaged in designing the protocol and will conduct the systematic map. Two of the authors (FCA and GSS) will undertake the literature search, screen publications for relevance, determine their eligibility, and document the results and the systematic map itself. The third author (AC) will act as a mediator when there is a lack of consensus between the reviewers during the eligibility assessment. Additionally, AC will collaborate with the other authors to review and refine the findings and the systematic map.

## Objective of the systematic map

This systematic map aims to collate scientific data on the presence of birds and mammals in agricultural landscapes and their adjacent areas of natural vegetation, considering the Brazilian Atlantic Forest (BAF).

### Primary question

What are the species of birds and mammals in agricultural fields and their adjacent areas of natural vegetation?

#### Sub-questions

What are the different species of birds and mammals in agricultural fields that undergo varying management practices within the BAF?

What is the contribution of nearby natural areas to the diversity of birds and mammals in agricultural fields?

### Definitions of the question components

The question components will be addressed using the PICO/PECO model.

Population (P): Birds and Mammals (including flying and non-flying mammals and New World marsupials).

Exposure (E): Agricultural Landscape in the BAF (e.g., perennial crop, annual crop, agroforest, tree plantations, and pasture) and its adjacent areas of natural vegetation (e.g. forests, natural fields, etc.).

Comparator (C): Diversity and abundance of birds and mammals in the agricultural landscapes (farmland, pasture, tree plantation, agroforest) in the BAF compared to adjacent natural areas.

Outcome (O): The species' occurrence in agricultural fields and adjacent natural areas according to abundance, diversity, guilds, habitats, crops, regions, and the number of studies for each case.

## Methods

The proposed systematic map will follow the standards and guidelines from the Collaboration for Environmental Evidence [15] and the ROSES reporting standards for systematic map protocols ([20] and ROSES, 2023) (see Additional file 1, 5, ROSES_Systematic Map Protocol.xlsx). In addition, as required by CEE standards, this protocol has been registered in PROCEED (https://www.proceedevidence.info/) as PROCEED-23-00166.

The literature search will be performed in English and Portuguese. In all databases, we will not limit the year of publication, performing searches up to the present time. All literature searches will be combined using Rayyan's systematic review tool [16].

### Search string and the comprehensiveness of the search

Search terms were developed to comprehend previously described PECO elements (e.g., Birds, Mammals, Brazilian Atlantic Forest, Agriculture, Farm, etc.). A preliminary pilot search was conducted using Google Scholar's Advanced Search feature to elaborate a search string for bibliometric databases such as Web of Science (WoS), Scopus, and PubMed. A preliminary screening string was employed to conduct the pilot search, details of which are provided in Additional file 2 (Benchmark studies_Birds and Mammals_Search String_revised.xlsx). This pilot search yielded more than 6000 publications. From this extensive collection, a dataset comprising 100 publications was selected and documented in Additional file 3 (Literature Eligibility Pilot_Consolidated.xlsx). The selection process for these publications was initially based on the scrutiny of titles; however, in instances where an article was considered relevant, a subsequent search for related articles was also undertaken.

A pilot screening and eligibility assessment was conducted to ascertain the eligibility of selected publications per the PECO model (see Eligibility criteria ahead in the text). This pilot exercise resulted in the identification of 24 eligible publications (see Additional file 3). Subsequently, 10 publications were designated as benchmark articles; these were selected based on their titles and citation indices. The benchmark publications were then screened in full text to identify keywords that cover elements of the PECO model. This procedure included terms related to population (e.g., "birds," "mammals," "avifauna"), exposure (e.g., "agriculture," "farm," "cropland"), and comparison (e.g., "Atlantic forest," "Atlantic rainforest"). After identifying the keywords and additional keywords recommended after the protocol's peer-review process, the string was used to determine the efficacy of retrieving all 10 benchmark publications within the WoS database. The initial test successfully located all benchmark publications, obviating the need for further refinement of the search string. The definitive search string is delineated in Table 1.

**Table 1:** Final search string

Database	Search string
Wos, Scopus, And Pubmed	(BIRD* OR MAMMAL* OR AVIFAUN* OR PRIMATE* OR RODENT* OR AVIAN OR MARSUPIAL* OR FAUN* OR OMNIVOR* OR CARNIVOR* OR FRUGIVOR*, GRANIVOR*OR INSECTIVOR* OR BAT* OR PASSERIN* OR CHIROPTER*) AND (AGRICULT* OR CROP* OR FARM* OR PASTURE OR MATRIX OR PATCH* OR CORRIDOR* OR “PROTECTED AREA” OR PLANTATION* OR FRAGMENT* OR TROPIC* OR ECOSYSTEM* OR AGROFOREST* OR “SECONDARY FOREST*” OR “FOREST REMNANT*” OR “AGRICULTURAL FIELD*” OR “PRIMARY FOREST*” OR ARABL* OR SYSTEM* OR PRACTICE* OR MANAGEMENT OR ORGANIC OR AGROECOLOG* OR "CONSERVATION AGRICULTURE" OR "PEST MANAGEMENT" OR BIOCONTROL OR "URBAN AGRICULTURE" OR TILL* OR ABANDONMENT OR SET-ASIDE OR FALLOW* OR "MIXED CROP-LIVESTOCK*" OR "INTEGRATED CROP-LIVESTOCK*" OR "DIVERSIFIED CROP-LIVESTOCK*" OR "VEGETATION STRIP*" OR "INSECT STRIP*" OR "FLOWER STRIP*" OR DIVERSIFICATION OR ROTATION OR "INTER-CROP" OR COVER-CROP*) AND (“ATLANTIC FOREST” OR “BRAZILIAN ATLANTIC FOREST” OR RIPARIAN OR SEMI-DECIDUOUS OR “BRAZILIAN ATLANTIC RAINFOREST*” OR NATIONAL PARK* OR TREE PLANTATION* OR PERENN* OR “PERENNIAL CROP*” OR NEOTROPIC*) AND NOT (CAATINGA OR CERRADO OR AMAZON OR PAMPA OR ARGENTINA) AND BRAZIL
Biblioteca digital brasileira	(AVE OR AVIFAUNA OR PASSERIFORME OR MAMÍFERO OR MASTO OR ROEDOR OR PRIMATA OR MARSUPIAL OR FAUNA OR INSETIVOR* OR CARNÍVOR* OR GRANÍVOR* OR FRUGÍVOR* OR ONÍVOR* OR MORCEGO OR QUIRÓPTERO) AND (AGRICULTURA OR AGRO* OR FAZENDA OR CULTIVO OR PLANTAÇÃO OR PAST* OR MATRIZ OR FRAGMENTO OR CORREDOR OR "ÀREA PROTEGIDA" OR TROPICAL OR AGROFLORESTA* OR ECOSISTEMA* OR "FLORESTA SECUNDÁRIA" OR "FLORESTA PRIMÁRIA" OR "FLORESTA REMANESCENTE" OR "CAMPO AGRÍCOLA" OR ARÁVEL OR SISTEMA OR PRÁTICA OR MANEJO OR ORGÂNIC* OR AGROECOLOG* OR "MANEJO DE PRAGAS" OR BIOCONTROLE OR "PLANTIO DIRETO" OR POUSIO OR ROTAÇÃO OR DIVERSIFICAÇÃO OR "COBERTURA VEGETA*" OR "AGRICULTURA URBANA" OR "FAIXA DE FLORES" OR "FAIXA DE INSETOS" OR "FAIXA DE VEGETAÇÃO" OR "CULTURA DE COBERTURA" OR "SISTEMA LAVOURA-PECUÁRIA" OR "INTEGRAÇÃO LAVOURA-PECUÁRIA" OR "LAVOURA-PECUÁRIA DIVERSIFICADA") AND ("MATA ATLÂNTICA" OR "FLORESTA ATLÂNTICA" OR PERENE OR "CULTIVO PERENE" OR NEOTROPICAL OR SEMIDECÍDUA* OR RIPÁRIA NOT CAATINGA NOT CERRAD* NOT AMAZÔNIA NOT PAMPA NOT AMAZON* NOT ARGENTINA)

The asterisk (*) is a wildcard, which will provide variations of a root word. For example, using the string agroforest* will return words such as agroforest, agroforests, and agroforestry. To search for exact phrases in the string above, we have used quotation marks “”, like the example “BRAZILIAN ATLANTIC RAINFOREST”

### Bibliographic databases

The literature search will be performed on three bibliometric databases: Web of Science Core Collection (WoS), Scopus, and PubMed.

### Web-based search engines/organizational sites

No additional search will be conducted on website search engines and organizational websites.

### Grey literature search

A grey literature search will consider thesis and dissertations only and will not include conference abstracts and posters. Searches will be performed in the “*Biblioteca Digital Brasileira*” database (https://bdtd.ibict.br/vufind/). We have translated the search string from English into Portuguese (see Table 1). After that, we performed a pilot test as follows:

- The Advanced Search option was selected, and the search string in Portuguese was inserted in the appropriate fields;
- Idiom—“no selection”. Nonetheless, since the search string was inserted in Portuguese, retrieving literature in Portuguese;
- The options “All fields” and “all terms” were selected to perform the search;
- The option “all document types” was selected;
- period of search—to perform the survey up to the present moment, we have selected the option “no limit”;
- The item option “Illustrations” was set as “no preference”;

After inserting the search string, the result indicated that translating the search string from English to Portuguese enabled us to perform the literature search according to the PECO model, and no further adaptation is required.

### Literature search update

After the publication of the systematic map protocol, we expect to conclude the literature search and publish the results within a year. Nonetheless, suppose this process takes longer, and a systematic map manuscript is not submitted after one year. In that case, an update on the literature search will be performed if the time between the last literature search exceeds one year after the publication of the protocol**.**

## Article screening and study eligibility criteria

**Fig. 2:**
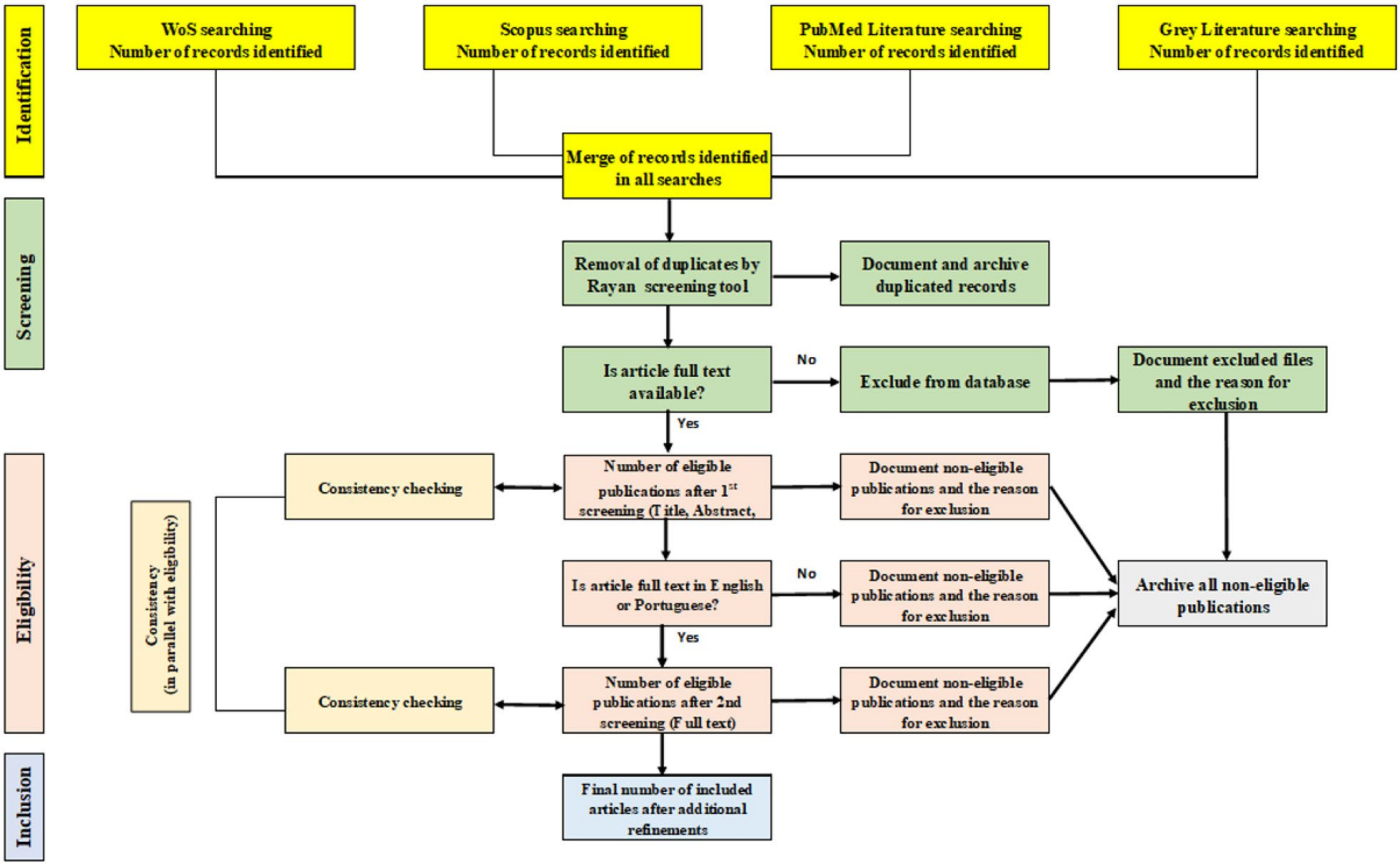
Summary of map protocol screening and eligibility strategy and consistency checking

The methodology employed for literature screening, eligibility, and consistency checking is delineated below (steps 1–3) and shown in Fig. 2. Two reviewers (FCA and GSS) will perform publication screening and eligibility. In parallel, consistency checking will be performed in both steps.

### Step 1: Screening strategy

Figure 2 provides a visual summary of the screening strategy employed in this systematic map. To facilitate the removal of duplicate entries from the search results across all databases (Web of Science, Scopus, and PubMed), we will utilize the systematic review software Rayyan [16]. Documentation will be maintained for all duplicate files identified and removed. After the deduplication process, we will verify the availability of the full text for each publication. Those publications for which the full text is not accessible will not advance to subsequent stages of review, and the rationale for their exclusion will be systematically recorded. As previously mentioned, the literature search will also take into account the following elements:

*Study type*: qualitative and quantitative studies, reviews, meta-analyses, modelling studies.

*Timeframe*: There will be no limited timeframe. The search will be conducted up to the present time.

*Language*: English and Portuguese.

### Step 2: Eligibility criteria

The literature search will be refined using the eligibility criteria delineated below. The PECO model has formulated these criteria, as described earlier in the text. Publications failing to satisfy these eligibility criteria will be excluded from further review stages. Records and detailed justifications for their exclusion will be maintained for all files deemed non-eligible.

*Eligible population*: birds, mammals (including flying and non-flying mammals and New World marsupials).

*Eligible exposure*: Brazilian Atlantic Forest (BAF) and Agricultural Landscape (e.g., perennial crop, annual crop, agroforest, tree plantations, pasture, and urban agriculture).

*Eligible comparator*: diversity and abundance of birds and mammals present in the agricultural landscape (farmland, pasture, tree plantation, agroforest) and its adjacent natural areas in the BAF.

*Eligible outcome*: Identify the species in agricultural fields and its adjacent natural areas according to abundance, guilds, habitats, crops, regions, and the number of studies for each case.

The assessment of eligibility will be conducted in a two-step process: (a) an initial appraisal of titles and abstracts, followed by (b) a subsequent evaluation of full texts. In the preliminary phase, should a reviewer encounter uncertainty regarding a publication's compliance with the selection criteria, the study will proceed for full-text review. The rationale for exclusion will be documented during the second screening phase. A list of excluded studies will be made available, accompanied by the reasons for their rejection.

### Step 3: Consistency checking

FCA and GSS will independently screen the same subset of articles according to the eligibility criteria described in Step 2. To evaluate the consistency between the reviewers, these reviewers will evaluate an initial sample of 10% of publications, and the outcome will be compared for consistency by estimating the percentage of agreement and by calculating Cohen’s Kappa (k) index [17], aiming for a k > 0.6, which indicates substantial agreement [18]. If the k index is below 0.6, reviewers will discuss the reasons for disagreement, record it, and decide eligibility. After that, the reviewers will screen an additional sample of 10% of the publications, and the Kappa index will be calculated again. This procedure will be repeated until Cohen’s Kappa is higher than 0.6. In cases where disagreement persists between the reviewers, a third reviewer will act as a mediator.

### Pilot test

To evaluate the efficacy of our strategy, a pilot test was conducted utilizing a database of 100 publications, including articles, book chapters, and theses. The results and details of this pilot test are documented in Additional file 3 (Literature Eligibility Pilot_Consolidated.xlsx).

A dataset of 100 publications was utilized to execute a pilot screening and eligibility assessment; the procedures evaluated this dataset delineated in Steps 1 through 3, as previously described. During the eligibility evaluation phase (Step 2), each publication underwent a review based on the criteria indicated in the questions presented below. The formulation of these questions was designed to verify the adherence of the publications to the PECO model. The details of this pilot can be found in Additional file 3 (Literature Eligibility Pilot_Consolidated.xlsx).

1. Does the publication include birds or mammals?
2. Was the study conducted in the BAF?
3. Was the study conducted in an agricultural landscape (including its adjacent natural areas)?
4. Does the publication measure occurrence?

The pilot test was executed independently by FCA and GSS. Initially, each reviewer scrutinized the title, keywords, and abstracts of the publications in light of questions 1 to 4. In this phase, if the answer was “No” to at least one of the four questions, the publication was deemed not eligible. Conversely, if the answer was “Yes” to most of the questions, but the reviewer was unsure about one, the publication was subjected to a comprehensive full-text review, as delineated in Fig. 2 under the 'eligibility step'. The complete text was evaluated against the same set of questions during this stage. A negative response to any question at this point once again rendered the publication ineligible. Throughout all stages, publications that failed to meet the eligibility criteria were excluded from the data extraction phase. The justifications for exclusion were recorded, and the non-eligible publications were subsequently archived.

The assessment outcome from each reviewer was also evaluated for consistency. The degree of agreement between reviewers was estimated through the Kappa statistic (Cohen’s k) [17]. This process resulted in a raw agreement of 81.6% and a Cohen’s k index of 0.6, which indicates moderate agreement. Instances where there was a lack of consensus on the eligibility of a publication were discussed by FCA and GSS to resolve whether the publication in question should advance to the data extraction phase. Upon agreement from both reviewers, the outcomes were consolidated, culminating in a total of 24 publications that were adjudged eligible. Additional publications deemed non-eligible were archived, and the reasons for exclusion were documented. At this point, no mediator between the two reviewers was needed.

### Reporting screening outcomes

When the literature search is complete, we will follow the Reporting for Systematic Evidence Synthesis (ROSES) guidelines by filling out the ROSES form for Systematic Maps Reports and the ROSES flow diagram. ROSES forms and diagrams are meant to ensure that the Systematic Map includes all necessary content required by the is present and described in detail (Headway et al. 2018). The form will help describe the methodology and report research synthesis and will also help interpret the data and draw conclusions, especially in identifying knowledge gaps.

A ROSES flow diagram will also be included in the final manuscript to help summarize the information required by the ROSES Map Report form. The flow diagram will also present the number of studies assessed and rejected at each screening stage and the number of rejected studies. Full-text screening will be recorded with the reason for rejection. The ROSES file will be uploaded and provided as supplementary material. The systematic map will discuss any deviations from the ROSES flow diagram.

### Study validity assessment

No study validity assessment will be completed.

### Data coding strategy

After the screening and eligibility evaluation, the selected articles will proceed to data extraction to gather data on species abundance in the agricultural landscape.

Table 2 and Additional file 4 (Pilot_Data Coding Strategy_Data Extraction.xlsx) indicate the details of the data coding strategy, indicating what the codebook will contain and how the information from the surveyed literature will be extracted*.*

**Table 2:** Data coding strategy

Variables	Description	Example^a^
ID_code	Identification code for each species extracted from each publication	BMA0001
Species/order	Genera and species name	*Zenaida auriculata*
Crop	Identify crop from each publication	Coffee, cacao
taxonomic_group	Studied group	(a) Birds (b) Mammals (large/medium-sized mammals, carnivores, primates) (c) Small mammals, bats
bio_organization	Indicate the focus of the study as defined by the authors	(a) Population = study is focused on more than one population of the same or different species (not identified as an assemblage or community study) (b) Community = all species of the determined area (c) Assemblage = sub-group of community
agricultural_system	Type of agricultural system	(a) tree_plant = tree plantations—eucalyptus, pinus, rubber trees, etc (b) crop_annual = annual crops—beans, soybeans, wheat, sugarcane, corn, rice, potato, cotton
ecosystem		(a) forest = forest (mixed forest, semideciduous, contiguous, fragment) (b) wetlands = (wetlands and floodplain areas)
CU_area	Describe the type of conservation unit (CU) from where the data was taken from	**(**a) inside_cu = inside of a legal CU area (b) outside_cu = outside of a legal CU area, including forest fragments
GPS_info	GPS information. If not available, indicate as NA	NA = no data or not specified
Region	Identify the municipality and/or state. If not available, indicate as NE	NE = not specified
Title	Publication's title	Biodiversity extinction thresholds are modulated by matrix-type
Publication ID	Publication identification. One for each publication	PBMA0001
Authors	author's names	Boesing A.L., Nichols E., Metzger J.P
source_title	Identify the Journal name or University (Grey Literature)	Ecography
Year	Year of publication	2018
Abstract	Publication abstract	Biodiversity extinction thresholds are abrupt declines in biological (…)
DOI	DOI number, if not available, indicates NA	10.1111/ecog.03365
study_type	Describe the type of the study	(a) Data papers (e.g., Ecology data papers) = compilation of studies without question (b) Secondary_data = study is based on secondary data from other databases (c) Empirical = scientific study with a question (collected in the field)

^a^A complete description of the codebook can be found in Additional file 4

We have also performed a pilot test on data coding and extraction, recorded in Additional file 4. In the pilot, we used the 10 benchmark publications to develop the search string and extracted data on species abundance for each crop.

### Consistency checking

For data extraction, FCA and GSS will independently extract data from all eligible studies. However, if the number of studies is large, only the primary reviewer (FCA) will extract the data, while the secondary reviewer (GSS) will subsequently check in a sample of 20% of the publications. Discrepancies in the data coding will be discussed within the team (FCA, GSS, and AC).

## Study mapping and presentation

The systematic map will provide a narrative and quantitative synthesis. The research gaps will be identified by analyzing the meta-data representation, which will be visually shown as tables or diagrams according to guilds, habitats, and crops. The data collected, meta-data, and codes will be reported in the final manuscript or as Additional files.

## Supplementary Information

**Additional file 1:** Roses systematic map protocol.xls

**Additional file 2:** Benchmark studies_Birds and Mammals_Search String_revised.xlsx

**Additional file 3:** Literature Eligibility Pilot_Consolidated.xlsx

**Additional file 4:** Pilot_Data Coding Strategy_Data Extraction.xlsx

**Additional file 5:** Roses systematic map protocol.pdf

## Data Availability

Not applicable.
