# Supplementary material for: What evidence exists on birds and mammals' biodiversity in the Brazilian Atlantic Forest (BAF) agricultural ecosystems? A systematic map protocol
Source: Environ Evid. 2024 Mar 16;13:5. doi: 10.1186/s13750-024-00327-4 (PMC11376101; doi:10.1186/s13750-024-00327-4)
Supplement: Supplementary file 5 — Additional file 5: Roses systematic map protocol.pdf [file 13750_2024_327_MOESM5_ESM.pdf]

number	Section / sub-section	Topic	Description	Further explanation	Checklist/Meta-data	Author response	Comments
1	Title	Title	The title must indicate that it is a systematic map protocol, and must indicate if it is an update/amendment: e.g. "A systematic map update protocol...".	The title should normally be the same or very similar to the review question.	Meta-data	systematic map	biodiversity in the Brazilian Atlantic Forest (BAF) agricultural ecosystems? A systematic map protocol
2	Type of review	Type of review	Select one of the following types of review: systematic map, systematic map update, systematic map amendment	See CEE Guidance on systematic mapping [1], and on amendments and updates [2]	Meta-data	systematic map	
3	Authors contacts	Authors contacts	The full names, institutional addresses, and email addresses for all authors must be provided.		Checklist	Yes	(1) Ana Paola Cione, Syngenta Proteção de Cultivos Ltda, Avenida Nações Unidas, 17007, 11th Floor, 04730-300 São Paulo, SP, Brazil (ana.cione@syngenta.com);<br>(2) Fabio Casallanovo, Syngenta Proteção de Cultivos Ltda, Avenida Nações Unidas, 17007, 11th Floor, 04730-300 São Paulo, SP, Brazil (fabio.casallanovo@syngenta.com);<br>(3) Gustavo Souza Santos, Syngenta Proteção de Cultivos Ltda, Avenida Nações Unidas, 17007, 11th Floor, 04730-300 São Paulo, SP, Brazil (gustavo.santos@syngenta.com);
4	Abstract	Structured summary	Background, the context and purpose of the review, including the review		Checklist	Yes	
5	Background	Background	Describe the rationale for the review in the context of what is already known. Protocol must indicate why this study was necessary and what it aims to contribute to the field.	A theory of change and/or conceptual model can be presented that links the intervention or exposure to the outcome.	Checklist	Yes	
6	Stakeholder engagement	Stakeholder engagement	(e.g. in the formulation of the question) must be described and explained		Checklist	No	internal reviews will be performed
7	Objective of the review	Objective	applicable).	questions are usually linked to sources of heterogeneity (effect	Checklist	Yes	and mammals in agricultural fields and their
8		components	intervention(s)/exposure(s), comparator(s), and outcome(s).	For other question types see [4,5]	Meta-data	components will	
	Methods						
9	Searches	Search strategy		Details regarding search strategy testing should be provided.	Checklist	Yes	
10		Search string	the string is formatted (e.g. Web of Science format)		Meta-data	WoS, Scopus and	Brasileira): (Ave OR Avifauna OR Passeriforme OR
11		databases	List languages to be used in bibliographic database searches.		Meta-data	Portuguese	
12		Languages – grey literature	based search engines.		Meta-data	Portuguese	
13		Bibliographic databases	Provide the number of bibliographic databases to be searched.		Meta-data	4	Brasileira
14		Web – based search engines	Provide the number of web – based search engines to be searched.		Meta-data	0	
15		Organisational websites	Provide the number of organisational websites to be searched.		Meta-data	0	
16		comprehensiveness of the search	strategy was assessed (i.e. list of benchmark articles).		Checklist	No	PECO elements, such as population (e.g. birds,
17		Search update	review.	performed more than two years prior to review completion.	Checklist	Yes	the time exceeds one year between the last search
18	inclusion criteria	Screening strategy	relevance/eligibility.		Checklist	Yes	Screening against inclusion/exclusion criteria.
19		Consistency checking	including the levels at which consistency checking will be undertaken and		Checklist	Yes	abstract and full text according to pre-defined
20		Inclusion criteria	articles/studies. These must be broken down into the question key		Checklist	Yes	flying and non-flying mammals and new world
21		Reasons for exclusion	reasons for exclusion.		Checklist	Yes	provided as supplemental material
22	Critical appraisal	Critical appraisal strategy	validity (including assessment of individual studies and the evidence base	Optional	Checklist	n/a	
23		Critical appraisal used in synthesis	synthesis.	Optional	Checklist	No	
24		Consistency checking	tested.	Optional	Checklist	n/a	
25	Data extraction	strategy	(potentially providing forms/data sheets (ideally piloted), list if variables		Checklist	Yes	
26	presentation	Narrative synthesis strategy	base in the form of descriptive statistics, tables (including SM database)	of their findings) must be avoided. May include a summary of the	Checklist	Yes	Supplemental Material
27		identification strategy	knowledge gaps (unrepresented or underrepresented subtopics that		Checklist	Yes	Evidence Synthesis (ROSES) guidelines, by filling
28		independence	articles to be considered within the review) in decisions regarding	should be prevented from unduly influencing inclusion decisions, for	Checklist	Yes	appears in the search, it will be considered due to
29	Declarations	Competing interests	review authors may have.		Checklist	No	The authors declare no competing interests

References

[1] James, K.L., Randall, N.P. and Haddaway, N.R., 2016. A methodology for systematic mapping in environmental sciences. Environmental Evidence, 5(1), p.7.

[2] Bayliss, H.R., Haddaway, N.R., Eales, J., Frampton, G.K. and James, K.L., 2016. Updating and amending systematic reviews and systematic maps in environmental management. Environmental Evidence, 5(1), p.20.

[3] Haddaway, N.R., Kohl, C., da Silva, N.R., Schiemann, J., Spök, A., Stewart, R., Sweet, J.B. and Wilhelm, R., 2017. A framework for stakeholder engagement during systematic reviews and maps in environmental management. *Environmental Evidence* , *6* (1), p.11.

[4] Collaboration for Environmental Evidence. 2018. Guidelines and Standards for Evidence synthesis in Environmental Management. Version 5.0. www.environmentalevidence.org/information-for-authors.

[5] Leeds Institute of Health Sciences. https://medhealth.leeds.ac.uk/info/639/information_specialists/1500/search_concept_tools. Accessed 12/11/2017.
